# The global COVID-19 vaccine surplus: tackling expiring stockpiles

**DOI:** 10.1186/s40249-023-01070-7

**Published:** 2023-03-20

**Authors:** Nguyen Khoi Quan, Nguyen Le My Anh, Andrew W. Taylor-Robinson

**Affiliations:** 1 College of Health Sciences, VinUniversity, Gia Lam District, Hanoi, 100000 Vietnam; 2 Faculty of Medicine, Imperial College London, London, SW7 2AZ UK; 3 Center for Global Health, Perelman School of Medicine, University of Pennsylvania, Philadelphia, PA 19104 USA

**Keywords:** COVID-19, SARS-CoV-2, Omicron, Vaccination, Immunization, Vaccine surplus, Vaccine hesitancy, Vaccine reluctance, Vaccine inequity

## Abstract

**Background:**

A global surplus of coronavirus disease 2019 (COVID-19) vaccines exists as a result of difficulties in aligning the demand and supply for vaccine manufacturing and delivery. World leaders have accelerated vaccine development, approval, production and distribution as a pragmatic approach to addressing the immediate public health challenges of the first two and a half years of the pandemic.

**Main body:**

The currently predominant, highly transmissible Omicron variant of severe acute respiratory syndrome coronavirus 2 has brought us closer to the threshold required to achieve herd immunity by greatly increasing rates of natural infection. Paradoxically, in parallel with rising vaccination levels in industrialized nations, this indirectly reduces the need for mass vaccine campaigns. Principal concerns that contribute to low vaccination rates which persist in several other countries, particularly of the Global South, are vaccine hesitancy and unequal access to vaccination. Social uncertainty fueled by fake news, misinformation, unfounded lay opinions and conspiracy theories has inevitably led to an erosion of public trust in vaccination.

**Conclusion:**

To address the current mismatch between supply and demand of COVID-19 vaccines, there should be a focus on three principles: decelerating vaccine production, increasing distribution across communities, and optimizing cost-effectiveness of distribution logistics. Slowing down and switching from large-scale production to effectively ‘made to order’ is a feasible option, which should be commensurate with management capacity. Transparent and evidence-based data should be widely and freely disseminated to the public through multimedia channels to mitigate miscommunication and conspiracy theories. Use of soon-to-expire stockpiles should be prioritized not only to enhance booster dose rollouts in adults but to expand immunization campaigns to children (especially those aged 5–11 years), subject to national approval. Future research should ideally aim to develop vaccines that only require basic, affordable storage and maintenance procedures as opposed to sophisticated and expensive protocols.

**Graphical Abstract:**

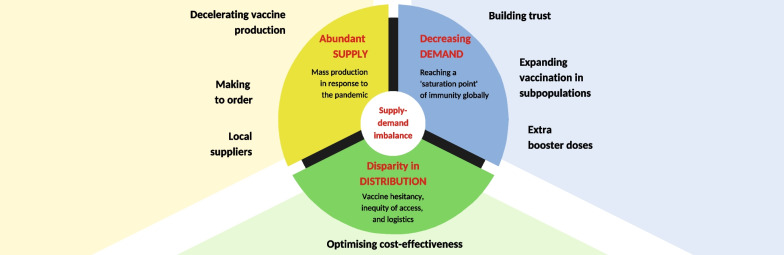

**Supplementary Information:**

The online version contains supplementary material available at 10.1186/s40249-023-01070-7.

## Background

**Fig. 1:**
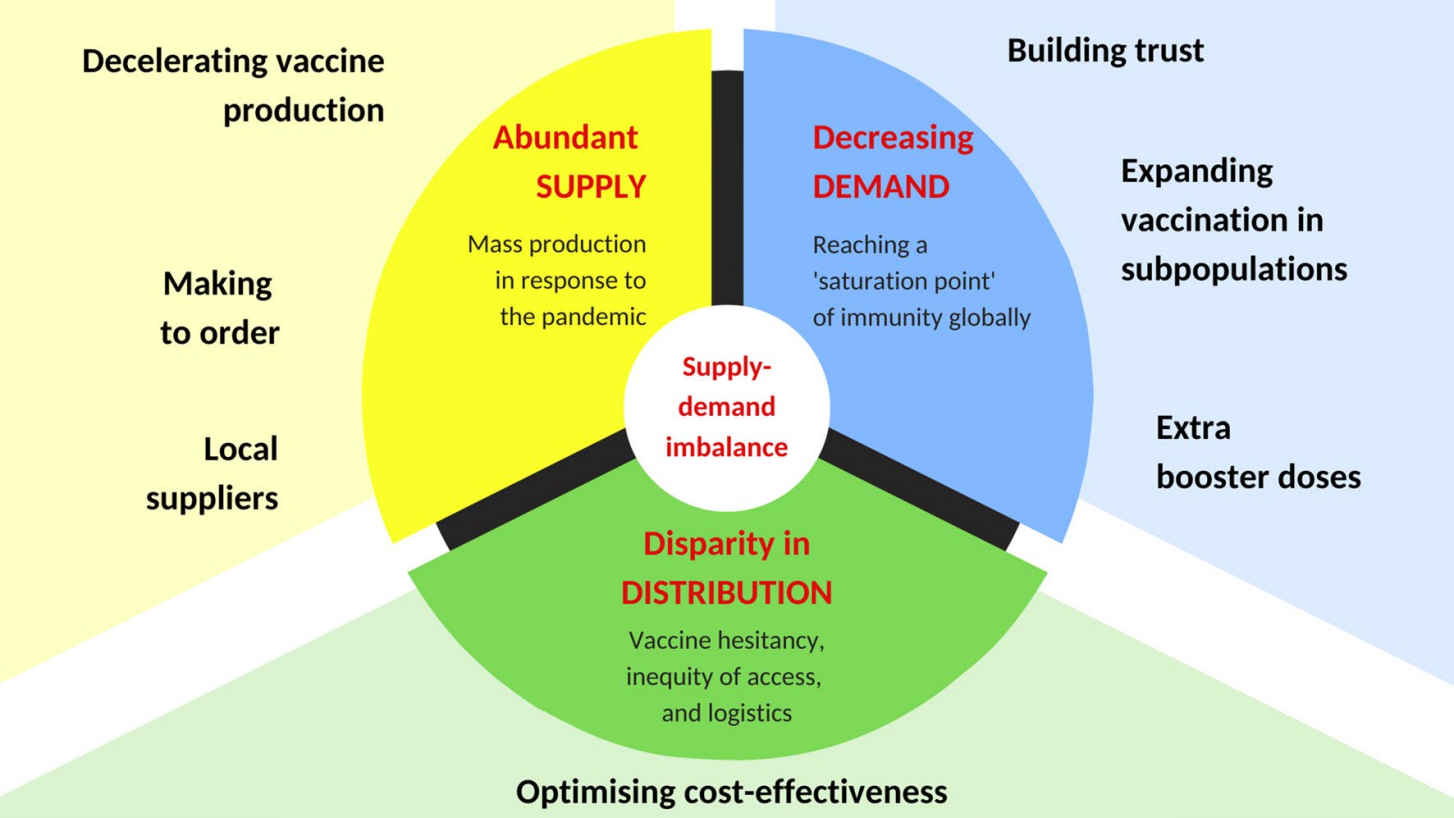
Tackling global COVID-19 vaccine surplus—at a glance

There is a surplus of coronavirus disease 2019 (COVID-19) vaccines globally [1, 2]. The estimated current wastage rate for COVID-19 vaccines is reported to be up to 30% [2]. This equates to approximately 5.6 billion doses, either opened and discarded or unopened but exceeded expiry before use. This inflated number reflects ongoing challenges in aligning the supply and demand for vaccine manufacturing and distribution. Here, we consider the current excess of COVID-19 vaccine stocks notwithstanding the need for vaccination, discuss plausible justification and suggest pragmatic solutions to avoid overstocking of COVID-19 vaccines around the world (summarized in Fig. 1).

## Reasons for excessive vaccine stockpiles

### Mass production in response to the pandemic

We are currently facing a significant challenge to public health resourcing in which many countries find themselves awash in COVID-19 vaccines [3–6]. Ever since the first reported case of COVID-19 in late 2019, the world has been drastically disrupted by the devastating clinical, social, and economic impacts of the pandemic. Enormous efforts continue to be made to explore effective treatments and prophylactic measures, including vaccination. By the end of 2020, it was reported that severe acute respiratory syndrome coronavirus 2 (SARS-CoV-2) was known to have infected more than 79 million people and caused over 1.7 million deaths worldwide, prior to the first vaccine being approved for emergency use by the World Health Organization (WHO) [7]. SARS-CoV-2 infection and COVID-19-related illnesses continue to affect all age groups, leading directly to more than 6.8 million fatalities across the globe as of January 31, 2023 [8].

From early 2021 all approved vaccines have been in mass production, with at least 18.6 billion doses procured to June 2022 [3], of which over 12.8 billion inoculations have been administered [9]. The global vaccination campaign is estimated to have saved nearly 20 million deaths in the first year alone and successfully reduced the rate of infection of COVID-19 [10]. By the end of January 2023, just over two-thirds (69.4%) of the world’s population have received at least one dose. However, overall figures disguise an alarming inequity between Global North and Global South (particularly in Africa and Middle East countries), exemplified by 76.7% of people living in the latter region yet to receive their first vaccination (Additional file 1) [9].

### Waning demand while reaching a ‘saturation point’

The dire consequences of COVID-19 worldwide have driven global leaders to focus on accelerating vaccine production as immunization is a favored and generally effective approach to infectious disease control. While supply is no longer a concern, the challenge now is one of lessening demand and unequal distribution. 2021 heralded a rapid expansion of vaccine manufacture in response to initial demand for first and second doses that were administered in many countries, primarily in the Global North. Yet, in 2022 there was a sharp decline in vaccine demand [4]. The emergence of the highly transmissible but reportedly less pathogenic SARS-CoV-2 Omicron variant of concern has strengthened herd immunity through a large rise in rates of natural infection. As more and more people contracted COVID-19 but experienced little or no clinical manifestations of infection with the Omicron variant, the attitude of the public started to change to consider the pandemic in a similar way to seasonal influenza (i.e., the “flu-ization” perspective) [11–13]. Thus, a growing number of previously vaccinated people are deciding not to receive a COVID-19 booster shot (typically, a third dose). While most are simply complacent, others argue that supplementary vaccination brings the risk—however small—of severe adverse outcomes, which is weighed against typically mild infection with Omicron following at least one dose of vaccine.

These attitudes act to further reduce vaccine rollout rates and worsen the supply-demand disparity, even though the global community reached a “tragic milestone” of one million deaths from COVID-19 in the first 8 months of 2022 [14]. Together with the present surge of cases in China, this serves as a reminder that the pandemic remains an international emergency [15]. In this context, it is axiomatic to point out that disease prevention is the target, while high vaccine rollout is simply a means to achieve this end. As SARS-CoV-2 will intrinsically continue to mutate, it is important to keep populations immunized to prevent further significant outbreaks with each new variant of concern. Booster doses are widely recommended for vulnerable groups, such as the elderly and immunocompromised, followed by lower priority groups of adults if 3–6 months have elapsed since receiving their last shot. The effectiveness of booster doses, particularly of the mRNA vaccine, in reducing the incidence and severity of infection has been documented [16–18].

### Disparity in distribution: vaccine protesters and inequity of access

Vaccine hesitancy is a major reason why COVID-19 vaccination levels rates remain suboptimal in poorly resourced regions, notably in the Global South [19]. Studies of vaccine reluctance in Vietnam [20, 21] and other low- and middle-income countries (LMIC) [22] have revealed the grounds on which false beliefs are based. Fake news, lack of authoritative information and misinformation about vaccination, particularly regarding its efficacy and possible adverse effects, have been instrumental factors in damaging public trust [20–22]. Social uncertainty generated by rumors and conspiracy theories has inevitably led to an erosion of faith in vaccination. Low trust in government, at national, regional and/or local level, is also a serious cause that needs to be addressed. Another issue that negatively impacts vaccine distribution is the inequity of accessibility [23, 24]. Those whose need is greatest have least access [19]. Most LMICs—those that do not have local vaccine production, effective distribution and/or reliable public healthcare—are still striving to immunize their citizens. Countries in sub-Saharan Africa are the prime example. Yet, at the same time several high-income countries (HICs) are experiencing COVID-19 vaccine abundance, with billions of doses surplus to requirements (Additional file 1) [1, 5]. Donating unused, in-date stocks could provide a solution to the global inequity of vaccine distribution, but only if hesitancy and concerns (especially towards the AstraZeneca vaccine) of recipients are dispelled [1].

## Solutions—“*omne trium perfectum*”, the rule of three

### Decelerating vaccine production and making to order

What is now considered “the rule of three” is an intuitive concept in many ancient belief systems that is best summed up by the Latin phrase *omne trium perfectum*: everything that is three is perfect [25]. This patterned reasoning continues to have practical significance today in, for instance, ideation, aesthetics, and communication, which here we apply to addressing the inequity of global vaccine distribution. Accordingly, in order to adjust the regional imbalance between supply and demand of COVID-19 vaccines, three actions should be prioritized—slowing down global vaccine manufacture, targeting distribution towards populations with poor rates of vaccine uptake, and improving supply chains, delivery infrastructures and storage logistics. The ongoing need for vaccination against COVID-19 will soon reach ‘saturation point’ as protective immunity is acquired among the global majority, notably in HIC, thanks to both natural infection and vaccination. Nonetheless, vaccine production should not be halted, even if temporarily, because COVID-19 remains potentially devastating to unvaccinated vulnerable individuals, while the various debilitating manifestations of post-COVID-19 syndrome (so-called “long COVID”) in some people remain to be fully elucidated [26]. Moreover, research and development into second generation vaccines that target Omicron or subsequent variants of concern is an important consideration for infection control and prevention while pandemic status persists. Downscaling the current mass production and adopting what would effectively be a ‘made to order’ strategy is a pragmatic solution.

Suppliers should make concerted efforts to align the shelf-life of vaccine stocks to match short-term vaccination needs, especially for countries where there is inadequate infrastructure to administer other nations’ redistributed surplus stocks with short expiry dates. Such a coordinated strategy would reduce wastage and could be implemented with the WHO-led COVID-19 Vaccine Delivery Partnership possibly acting as an intermediary [5, 6]. However, it is recognized that suppliers are mostly private companies that compete fiercely, so they may not cooperate to achieve such alignment. Another option is for LMIC to import large-volume frozen stock solutions that may be prepared on-site to meet local demand by thawing, aliquoting into vials, packaging and distributing only as and when required [27]. Plenty of pharmaceutical and biotechnology facilities at regional and national levels are accredited to perform a similar preparatory function with supplied bioproducts (e.g., insulin, antibiotics, monoclonal antibodies), which could turn their attention to this task with reasonable comfort. These local distributors would help to dramatically reduce the ‘vial-to-needle time’ by cutting the often-long delay for transportation and logistics of vaccine stockpiles. Local production—which is high on the political agenda—has been achieved in some countries, notably India and China [28], but is beyond the scope of most non-industrialized nations. Yet, by a combination of local preparation and distribution, if not production per se, various supply options should increasingly be able to match community level needs for vaccination in LMIC.

### Ramping up vaccine rollout

The ill-informed preference for a vaccine produced by one pharmaceutical company over another, matching first and booster vaccine formulations to optimize immunization efficacy, and an increasing expectation to update variant-specific vaccines, combine to compound issues of community non-compliance and make it an intricate task to achieve threshold levels of mass vaccination in LMICs. Building trust is key to fostering strong public engagement with vaccine rollout campaigns. Open access to information disseminated online makes it easy for conspiracy theorists to pass misinformation as factually correct. National public engagement programs to support COVID-19 immunization should not only aim to improve vaccine coverage, but also to ameliorate the groundless stigmatization of vaccination. Transparent and evidence-based data should be posted by a country’s national health ministry or public health authority on its official COVID-19 portal, then widely and freely circulated via government-approved multimedia channels. Dissemination by such trustworthy bodies will be generally accepted at all levels of society and help to counter miscommunication, discourage fake news, and suppress conspiracy theories [29].

A recent evaluation of efficacy and safety of COVID-19 mRNA vaccines has indicated their suitability to administer to children aged 5–11 years old [30]. This will go a long way towards alleviating parents’ concerns about side effects and strongly supports expanding immunization of this previously unvaccinated subpopulation in most countries. Nevertheless, further evidence from large cohort systematic studies that yield reliable data in favor of using vaccine types other than mRNA among this age range is essential to accelerate vaccine rollout. This is because a paucity of expert advice on this issue leaves many parents hesitant to provide consent to immunize their children due to unsubstantiated concerns over adverse reactions and potential long-term effects of COVID-19 vaccination [21, 31]. Given that vaccination-induced immunity to SARS-CoV-2 is temporary, there is a risk that more virulent and/or more transmissible variants could emerge. Administering extra booster inoculations (third, fourth doses, and so on) would safeguard against a major future outbreak by heightening immunity at a population level. There is an onus on each country to maintain a vaccine stock that is not excessive so as to create wasted surplus—as has occurred previously in many HIC—but rather sufficiently flexible to meet the supply and demand of the public health threat posed by SARS-CoV-2 at different times.

### Tackling the issue at scale: optimizing cost-effectiveness

Cost-effectiveness is a primary concern when vaccine surplus is considered on an international scale. COVID-19 vaccines have a short shelf life, and some require special storage conditions, making it challenging to transport to and administer in remote or poorly resourced locations. In order to maintain the vaccine with minimal degradation, research should focus on developing formulations that need only readily available storage facilities and inexpensive maintenance protocols rather than sophisticated equipment and expensive procedures. At present, some vaccines need to be refrigerated between 2 °C and 8 °C, while others require deep freezing at ultra-low temperatures, i.e., − 70 °C or lower. Manufacturing vials that contain fewer vaccine doses and/or extending the vaccine's shelf life can be another research priority [32]. For example, the Pfizer vaccine is currently packed as a six-dose aliquot per vial, for which there is a short deadline for use once opened. Hence, countless doses were discarded during the first global rollout due to ill-prepared and uncoordinated vaccination programs. Even with advanced registration, for multiple reasons a significant proportion of people may not attend for vaccination. Without prior cancellation of an appointment the lack of a replacement participant means that the unused dose is usually wasted. It should be feasible for hospitals and clinics within a given referral area to evaluate how best to share vaccine resources and to manage logistics both efficiently and effectively. By sharing instead of owning, the expense incurred by each practice center will decrease accordingly.

## Conclusions

The various authorized COVID-19 vaccines have collectively proved their effectiveness in reducing the severity and spread of SARS-CoV-2 as the world now adjusts to what is termed a ‘new normal’. By the end of January 2023, 69.4% of the world's population have received at least one dose of vaccine. Yet, in LMIC vaccination coverage plummets to barely more than a third of this total, 26.4%, leaving the remaining three-quarters of their population entirely unprotected [9]. Coupled with the current high rates of vaccine wastage, these sobering statistics demand immediate action from governments and the pharmaceutical industry worldwide. As we pass the third anniversary of the start of the COVID-19 pandemic, it is critical to ensure a balance between demand and production, as well as efficient, equitable vaccine distribution globally.

## Supplementary Information

**Additional file 1. **COVID-19 vaccination progress across the world (Retrieved from reference [9] on 26 January 2023).

## Data Availability

Not applicable.

## Abbreviations

COVID-19: Coronavirus disease 2019
SARS-CoV-2: Severe acute respiratory syndrome coronavirus 2
WHO: World Health Organization
LMIC: Low- and middle-income countries
HIC: High-income countries
