# Supplementary figures and images for: The global COVID-19 vaccine surplus: tackling expiring stockpiles
Source: Infect Dis Poverty. 2023 Mar 20;12:21. doi: 10.1186/s40249-023-01070-7 (PMC10025780; doi:10.1186/s40249-023-01070-7)

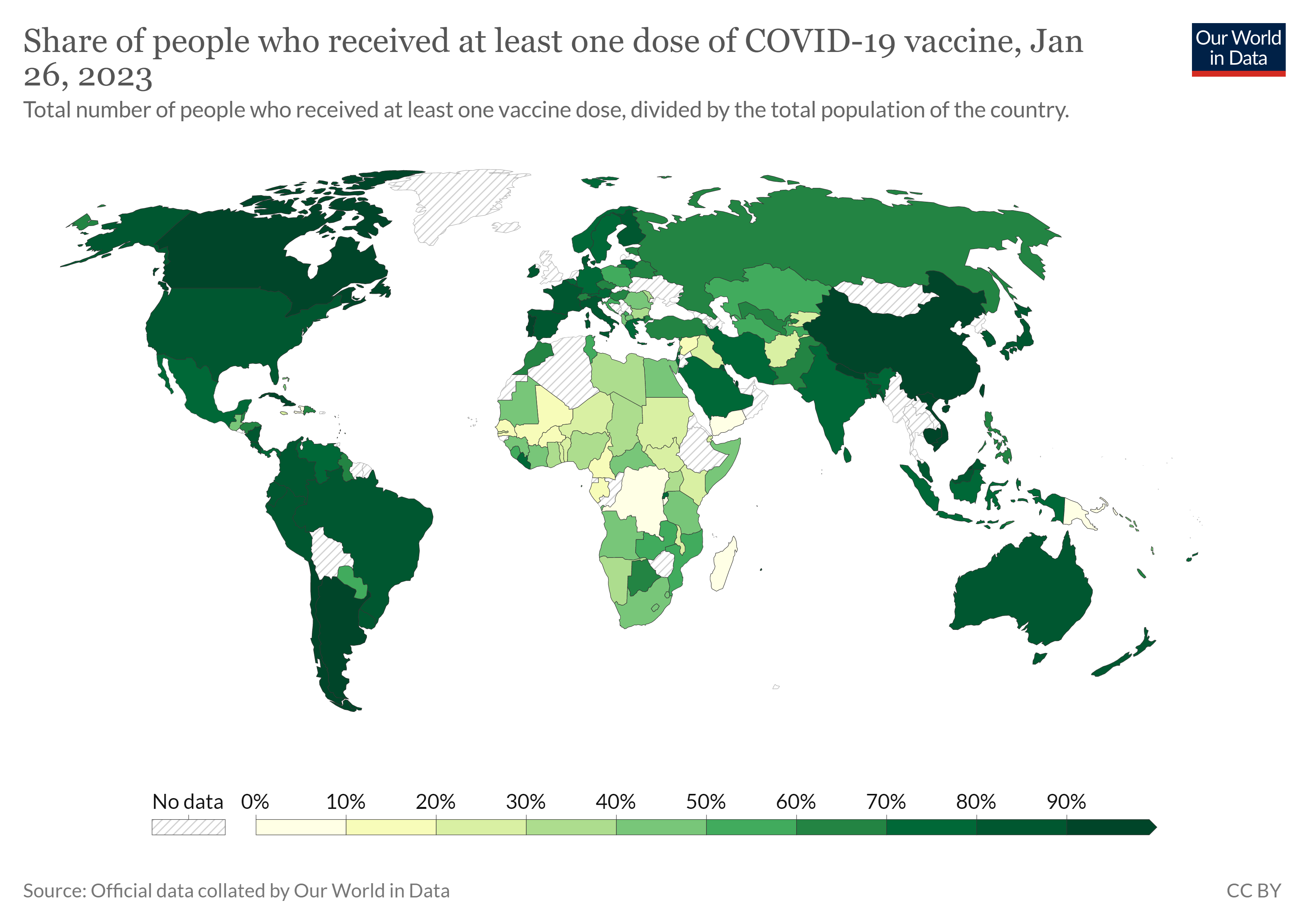

Supplement: Supplementary file 1 — Additional file 1. COVID-19 vaccination progress across the world (Retrieved from reference [9] on 26 January 2023). [file 40249_2023_1070_MOESM1_ESM.png]
